# Severe pulmonary involvement and elevated interferon-stimulated genes expression in two siblings with a novel *C1QB* pathogenic variant

**DOI:** 10.70962/jhi.20250128

**Published:** 2026-01-22

**Authors:** Clément Triaille, Guilhem Cros, Andrei-Bogdan Gorgos, Hélène Manganas, Anaïs Nombel, Eric Rich, Philippe Romeo, Sébastien Viel, Hugo Chapdelaine

**Affiliations:** 1 Department of Pediatrics, Pediatric Rheumatology and Immunology, CHU Sainte-Justine, Université de Montréal, Montreal, Canada; 2 Pôle de Pathologies Rhumatismales Systémiques et Inflammatoires, Institut de Recherche Expérimentale et Clinique, Université Catholique de Louvain, Brussels, Belgium; 3 Department of Immunology and Allergology, Centre Hospitalider de l'Université de Montréal, Université de Montréal, Montreal, Canada; 4 Department of Radiology, Centre Hospitalider de l'Université de Montréal, Université de Montréal, Montreal, Canada; 5 Department of Pneumology, Centre Hospitalider de l'Université de Montréal, Université de Montréal, Montreal, Canada; 6 Immunology Laboratory, Hôpital Lyon-Sud, Hospices Civils de Lyon, Lyon, France; 7 Department of Rheumatology, Centre Hospitalider de l'Université de Montréal, Université de Montréal, Montreal, Canada; 8 Department of Pathology, Centre Hospitalider de l'Université de Montréal, Université de Montréal, Montreal, Canada; 9 ARTEMIS Platform, Hôpital Edouard Herriot, Hospices Civils de Lyon, Lyon, France

## Abstract

Two siblings with a novel *C1QB* variant presented with severe pulmonary vasculitis and elevated interferon-stimulated gene expression. The findings suggest that hereditary C1q deficiency can manifest with life-threatening lung involvement driven by dysregulated type I interferon signaling.

## Teaser

We report vasculitis and interstitial lung disease in two siblings with a novel C1QB pathogenic variant. Patients responded to rituximab + tacrolimus or baricitinib. This report broadens the genotypic and phenotypic landscape of C1Q deficiency and extends on the similarities with other type 1 interferonopathies.

Hereditary C1Q deficiency is a very rare inborn error of immunity caused by biallelic pathogenic variants in *C1QA*, *C1QB*, or *C1QC*, leading to impaired classical complement pathway activity. Patients typically present in early childhood with a phenotype clinically indistinguishable from sporadic forms of systemic lupus erythematosus (SLE), although with a relatively lower frequency of renal involvement and high susceptibility to invasive encapsulated bacterial infections and central nervous system inflammation (1). Here, we report two patients with a novel *C1QB* pathogenic variant who both presented with early-onset SLE disease and later developed unusual inflammatory lung involvement.

**Figure 1.**
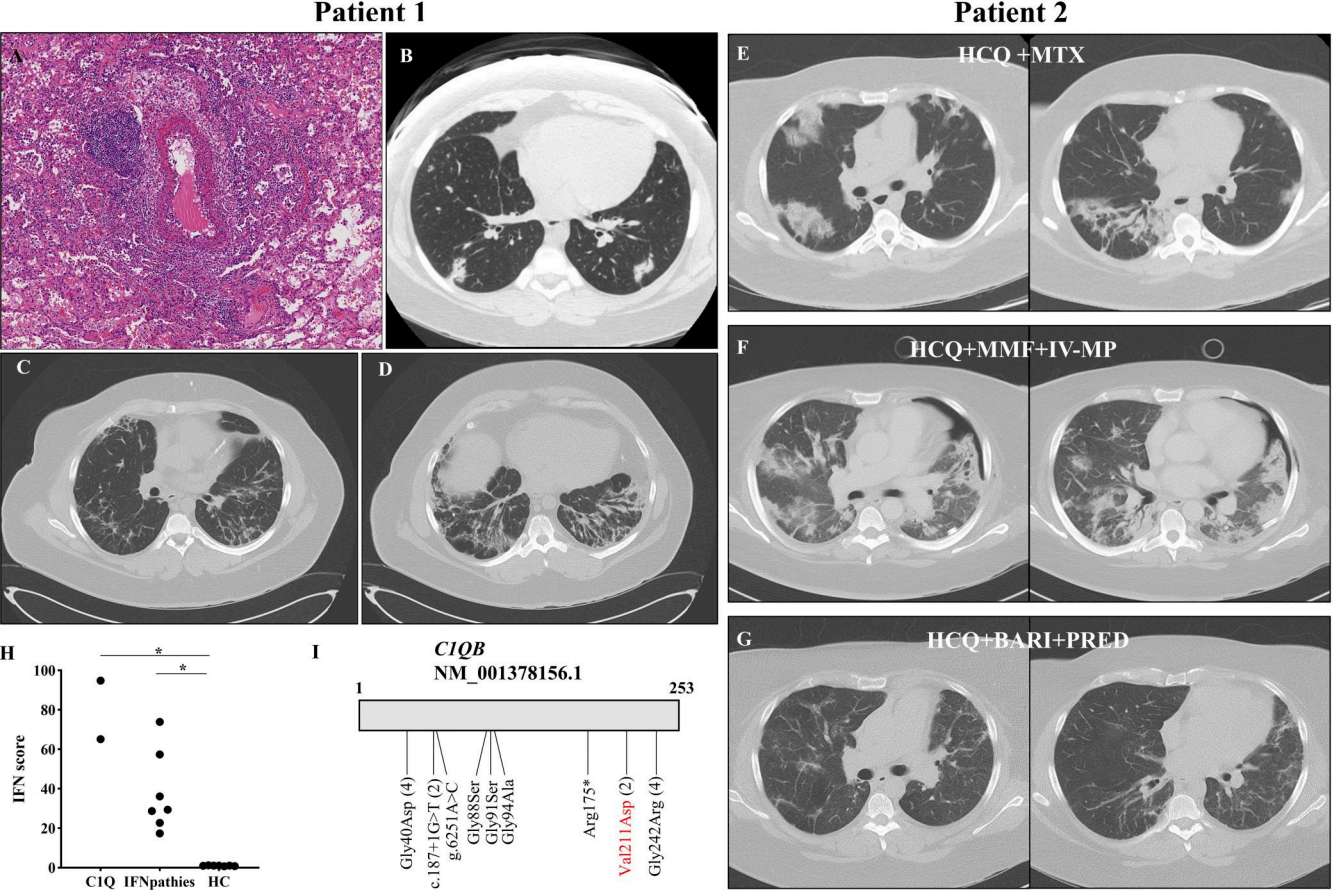
**Pulmonary vasculitis and fibrosing ILD in C1Q deficiency. (A)** Lung biopsy showing mild bronchiolitis and medium vessel vasculitis with fibrinoid necrosis. **(B)** Chest CT images at onset of hemoptysis in patient 1 (11 years of age) showing multifocal, well-defined pulmonary opacities. **(C and D)** Chest CT scan at last follow-up demonstrating significant residual ILD, with ground-glass opacities and reticular and cystic changes predominant in both lung bases. **(E)** Chest CT scan suggestive of organizing pneumoniae in patient 2: multiple foci of parenchymatous opacities in both lungs with reversed halo sign and air bronchogram in the right inferior lobe. **(F)** Chest CT scan 3 mo after (E), during intensive care unit admission, showing progression of bilateral alveolar opacities. Pneumothorax secondary to lung biopsy procedure. **(G)** Chest CT scan 1 year after images in F. demonstrating significant improvement of multifocal parenchymatous opacities. Ongoing immune-suppressive therapies are shown on the top of each panel: BARI, baricitinib; IV-MP, intravenous methylprednisolone; HCQ, hydroxychloroquine; MTX, methotrexate; PRED, prednisone. **(H)** IFN score computed based on normalized expression of type-1 ISG in peripheral blood in our patients (C1Q), other canonical type 1 interferonopathies (IFNpathies: Aicardi-Goutières syndrome [*n* = 3], Singleton–Merten syndrome [*n* = 2], and SAVI [*n* = 2]), and healthy controls (HC). The adjusted P values of Kruskal–Wallis test are shown on the graph (*: <0.05). **(I)** Schematic representation of the reported variants in *C1QB* in *n* = 15 patients in the literature and our two patients (shown in red). Of note, the transcript of reference used for this figure is NM_001378156.1; therefore, the variant of our patients is p.Val211Asp (which is equivalent to p.Val213Asp using NM_000491.3 as reference). When a variant has been reported in >1 patients, the total number of patients is shown between brackets. Figure created with the help of https://franklin.genoox.com/ for variant nomenclature and ProteinPaint for variant representation (https://proteinpaint.stjude.org/).

Patient 1 is a boy of French Canadian origin who developed a photosensitive rash and buccal ulcers at 7 years of age. Investigations revealed positive antinuclear autoantibodies (ANA, 1:160), autoantibodies against Ro/SSA and Sm, with negative anti-dsDNA; skin biopsy was notable for a vacuolar dermatitis with perivascular inflammatory infiltrate (no immunofluorescence performed). C3 and C4 values were normal, but classical complement pathway activity and C1Q were undetectable (respectively, 0% [reference value: 69–129%] and <27 mg/L [reference value: 197–277]). A novel homozygous variant in *C1QB* was identified (through Sanger sequencing of *C1QA*, *C1QB*,* C1QC*, *C1R*, and *C1S* genes, Fulgent Diagnostics): c.638T>A, p.Val213Asp (NM_000491.3, which is equivalent to p.Val211Asp using the canonical transcript NM_001378156.1 as reference). This variant is found at a very low frequency in the general population (gnomAD v4.1.0 allele frequency: 6.20e-7; no homozygote) and is predicted to be damaging by most in silico prediction tools combined annotation dependent depletion (CADD score: 25.6; PolyPhen-2: 0.995, rare exome variant ensemble learner [REVEL]: 0.908; AlphaMissense: 0.974 [likely pathogenic]). The patient was first treated with hydroxychloroquine with a favorable response. At age 9 years, he presented a meningitis due to *Neisseria meningitidis*. Severe hemoptysis first occurred at age 11 years. Multifocal opacities were notable on chest computed tomography (CT). Lung biopsy showed fibrinoid necrosis in small and intermediate vessels, with rare intravascular thrombi, perivascular lymphocytic inflammation, and signs of intra-alveolar hemorrhage. Mild bronchiolitis was also noted (Fig. 1, A and B). Infectious investigations returned negative. The patient was successfully treated with steroids and mycophenolate-mofetil (MMF). 2 years later, pulmonary relapse occurred. A second biopsy was mainly remarkable for milder signs of vasculitis (as described above) and apparition of significant interstitial fibrosis. Steroids and cyclophosphamide (six doses) were administered, with MMF as maintenance therapy (given rapid onset of cytopenia after azathioprine initiation). Replacement regimen of intravenous immunoglobulins was initiated for secondary hypogammaglobulinemia. A third relapse of pulmonary inflammation occurred at 20 years of age; the immunosuppressive regimen was therefore switched to rituximab and tacrolimus. The patient is currently 28 years old, and no other major event related to C1Q deficiency has occurred under this therapy and low-dose prednisone. At last follow-up, there was significant pulmonary fibrosis and interstitial lung disease (ILD) with a restrictive syndrome (forced vital capacity [FVC] 48% of predicted values) (Fig. 1, C and D).

Patient 2 is the younger sister of patient 1 and was diagnosed during familial screening with the same homozygous pathogenic variant in *C1QB*, undetectable C1Q, and classical complement pathway activity. In addition, ANA were positive with positive anti-Ro/SSA and normal C3 and C4 values. Mucocutaneous involvement appeared at 10 years of age and was successfully managed with hydroxychloroquine and penicillin prophylaxis until age 26 years, when a mucocutaneous flare and dyspnea occurred. Initial pulmonary imaging was normal, and methotrexate was introduced. Due to progressive respiratory symptoms, a control imaging showed the apparition of organizing pneumoniae (Fig. 1 E). Methotrexate was discontinued for a suspicion of lung toxicity. Yet dyspnea further progressed, leading to severe respiratory failure requiring intensive care unit admission (Fig. 1 F). Broad infectious investigations were negative, and an attempt of transbronchial lung biopsy was not contributive and complicated by pneumothorax. The patient was initially treated with high-dose steroids and MMF. Necrotizing pancreatitis developed shortly after MMF initiation, suggesting drug toxicity or uncontrolled disease. Therefore, MMF was replaced by Janus kinase (JAK) inhibitor (tofacitinib 10 mg daily, then baricitinib 4 mg daily to reduce pharmacological interactions). The patient was discharged 4 mo after admission. 1 year later, prednisone has been tapered to 4 mg daily, with baricitinib 4 mg daily and hydroxychloroquine. Chest imaging has significantly improved (Fig. 1 G), but there is significant structural damage and persistent restrictive syndrome (FVC 42% of predicted value).

We tested the expression of six interferon (IFN)-stimulated genes (ISG)—*SIGLEC1*, *IFI27*, *IFI44L*, *IFIT1*, *ISG15*, and *RSAD2*—in the peripheral blood of both patients at the latest follow-up (under therapy, without evidence of active disease) using NanoString technology (Hôpital Lyon-Sud). We computed a type 1 IFN signature, as published previously (2). Both displayed significantly increased ISG expression, comparable to other canonical type 1 monogenic interferonopathies (94.8 and 65.1 for patient 1 and 2, respectively [healthy control values < 1.5]) (Fig. 1 H).

We report on two siblings harboring a novel pathogenic variant in *C1QB*, which is amongst the rarest genes causing hereditary complement deficiency syndromes (<15 patients reported) (Fig. 1 I) (1). After an initial presentation with early-onset mucocutaneous SLE manifestation, both patients have experienced severe pulmonary involvement: recurrent episodes of small vessels pulmonary vasculitis and ILD in patient 1 and organizing pneumoniae in patient 2.

Several limitations should be considered in our report. Although the *C1QB* variant was predicted to be damaging in silico, extremely rare in the general population, and associated with undetectable C1Q protein and C100 activity in both homozygous individuals, its pathogenicity has not been mechanistically proven (e.g., by phenotypic rescue with complementation). Next, only a limited targeted gene panel sequencing has been performed at diagnosis. Therefore, one cannot formally exclude the possibility that the pulmonary phenotype might result from a second, undiagnosed genetic disorder. However, known monogenic conditions leading to ILD are rare and unlikely to present as childhood-onset alveolar hemorrhages with response to immune-suppressive therapy (with the notable exception of other type 1 interferonopathies such as coatomer protein complex subunit α [COPA] and STING-associated vasculopathy with onset in infancy [SAVI] syndromes). It is worth mentioning here that antineutrophil cytoplasmic antibodies, antiphospholipid antibodies, myositis-associated autoantibodies, and broad infectious workup (including fungal cultures and PCR for opportunistic pathogens on bronchoalveolar lavage) were negative in both patients.

To the best of our knowledge, lung involvement has never been reported in C1Q deficiency (for a total of ∼90 genetically confirmed patients [1]), in contrast with other canonical type 1 interferonopathies (such as COPA or SAVI syndromes). However, susceptibility to develop pulmonary vascular inflammation has been shown in *C1Q-*deficient mice (3). Thus, this pulmonary manifestation reported for the first time in C1Q-deficient patients from the same family might result from their specific *C1QB* variant and/or from the interaction with other genetic modifiers and/or from similar environmental exposures.

Two additional observations in these patients deserve to be underlined. Early-onset mucocutaneous disease is a typical initial manifestation of C1Q deficiency, which may respond favorably to first-line therapy; however, severe, refractory, and potentially life-threatening manifestations are not uncommon in the disease course (4). Consequently, hematopoietic stem-cell transplantation is increasingly considered as an early therapeutic intervention, with the potential to restore C1Q activity, reduce ISG overexpression, and achieve drug-free remission (1, 4, 5). Finally, patient 2 has favorably responded to JAK inhibitor, which is increasingly used in deficiency of the C1qrs complex (4). Overall, this report broadens the genotypic and phenotypic landscape of C1Q deficiency and extends on the clinical similarities with other well-characterized type 1 interferonopathies, providing further support to include this disorder within the spectrum of type 1 interferonopathies. Therefore, the potential benefit of direct type 1 IFN targeting (such as anifrolumab) deserves further investigation in C1Q deficiency (under consideration in our patients).

## Consent to participate

Informed consent for publication was obtained from the patients.

## Data Availability

Access to more detailed data can be discussed on an ad hoc basis following contact with the corresponding author.
